# Kinetic Theory Approach to Modeling of Cellular Repair Mechanisms under Genome Stress

**DOI:** 10.1371/journal.pone.0022228

**Published:** 2011-08-09

**Authors:** Jinpeng Qi, Yongsheng Ding, Ying Zhu, Yizhi Wu

**Affiliations:** 1 College of Information Science and Technology, Donghua University, Shanghai, People's Republic of China; 2 CSIRO Plant Industry, Urrbrae, South Australia, Australia; The University of Hong Kong, Hong Kong

## Abstract

Under acute perturbations from outer environment, a normal cell can trigger cellular self-defense mechanism in response to genome stress. To investigate the kinetics of cellular self-repair process at single cell level further, a model of DNA damage generating and repair is proposed under acute Ion Radiation (IR) by using mathematical framework of kinetic theory of active particles (KTAP). Firstly, we focus on illustrating the profile of Cellular Repair System (CRS) instituted by two sub-populations, each of which is made up of the active particles with different discrete states. Then, we implement the mathematical framework of cellular self-repair mechanism, and illustrate the dynamic processes of Double Strand Breaks (DSBs) and Repair Protein (RP) generating, DSB-protein complexes (DSBCs) synthesizing, and toxins accumulating. Finally, we roughly analyze the capability of cellular self-repair mechanism, cellular activity of transferring DNA damage, and genome stability, especially the different fates of a certain cell before and after the time thresholds of IR perturbations that a cell can tolerate maximally under different IR perturbation circumstances.

## Introduction

Generally, a biological system consists of from a few copies to millions of different components with specific interactions. Especially, as a unit of a bio-system, a cell also consists of a large number of active molecules, such as, DNA, mRNA, protein etc [1]. In response to genome stress under acute perturbations from outer environment, a cell can start its internal self-defense mechanism triggered by complicated interactions among these “active particles” [2], [3]. Under acute IR, for example, DNA in a normal cell is broken down, and the Double Strand Breaks (DSBs) occur stochastically. Fortunately, a normal cell can trigger its self-repair mechanisms to fix DNA damage induced by external IR perturbation. Repair Protein (RP), a kind of repair enzyme, can bind into the nascent DNA ends and further synthesize DSBCs [4], [5]. As a main signal source of transferring genome stress, DSBC can relay DNA damage to downstream genes and their signal pathways. Especially, P53, a vital anti-cancer gene, can be activated by DSBCs transferring, and active P53 can prompt its downstream genes, and further control the cell cycle arrest and abnormal cell apoptosis in order to eliminate lethal genome damage or deregulated proliferation [6], [7].

The combined approaches of control theory, system biology, and bioinformatics can stimulate new ideas to investigate the complicated mechanisms of biological system, which provide a good link between the diverging areas of biomedicine and mathematics [8]. For instance, as a novel mathematical approach, KTAP can be used in modeling the overall system by evolution equations corresponding to the dynamics of all their elements [9]. By this approach, a biological phenomenon can be described as the evolution of the dynamics of several interacting modules [10]. In some cases, biological system can be characterized by a discrete, rather than continuous, biological state. The description of bio-system by methods of mathematical kinetic theory essentially means defining the microscopic state of these interacting molecules and the distribution function over the above state [10].

KTAP is motivated not only by applied mathematicians, but also by researchers in the field of biological sciences. An important hint on the use of methods of kinetic theory and non-equilibrium statistical mechanics is given by Bellomo and Forni [11]. The methodology of statistical mechanics and kinetic theory to model complex biological system is capturing the attention of applied mathematicians, as documented by Deutsch and Dormann [12]. Degond, Pareschi, and Russo [13], and Cercignani and Gabetta [14], give an account of the surveys related to the application of the methods of kinetic theory to model complex systems. Moreover, KTAP is applied in various fields of social and life sciences, e.g. modeling vehicular traffic flow [14], [15], and social behaviors of interacting individuals [16], especially, multi-cellular systems [1], [12], [17], and tumor-immune system competition etc [18]–[20].

Recently, several mathematical frameworks have been proposed to represent the stochastic dynamics of DSB repair process, such as Monte Carlo simulation methods in [21], as well as ordinary differential equations models in [22]–[26]. To further investigate cellular self-repair mechanisms under acute IR from outer environment, a model of DNA damage generating and repair process is proposed at single cell level via the approach of KTAP. Compared with the previous work done mentioned in [22]–[26], KTAP is efficient in modeling the bio-systems of interacting active particles where the interactions among the active molecular particles modify their individual state and generate proliferating and destructive events. Also, this approach can capture the dynamics of the CRS in a better way, and help to deeply investigate and analyze the complicated cellular self-defending mechanism in response to acute perturbation environments.

This paper is organized into four parts. Section 2 aims at providing a brief survey of the mathematical framework relating to an open bio-system of active particle, describing the profile of CRS via the approach of KTAP. Section 3 presents the complex interactions between active particles of two sub-populations, and implements the processes of DSBs and RP generation, DSBCs synthesis, and toxins accumulation, respectively. Section 4 illustrates the dynamic kinetics of cellular self-repair mechanism by using the simulation platform of MATLAB7, and roughly analyzes the capability of DNA damage repair process, cellular activity of transferring DNA damage, and genome stability, especially, the different fates of a certain cell are analyzed before and after the time thresholds of IR perturbations that a cell can tolerate maximally under different perturbation circumstances.

## Methods

### Mathematical frameworks for an open bio-system

KTAP describes dynamic evolution of the probability distribution over the microscopic state, called activity, of several interacting entities called active particles [10]. The equation which models the evolution is derived by a conservation balance in the elementary volume of the space of the microscopic states, where the inlet and outlet flows are determined by interactions between active particles [27]. Based on existing approach mentioned in [28], the content of this section aims at providing a brief survey of the mathematical frameworks relating to an open bio-system of active particles, as well as modeling how an open bio-system of active particles interacts with the ‘acute perturbation agent’ from outer environment.

Consider an open bio-system constituted by a large number of active particles organized into *n* populations labeled with the subscripts *i* = 1,…,*n*. The activity of the particles are represented by a discrete variable *u* belonging to the set 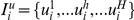, with components 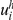, where *h* = 1, . . . , H. Consider the case in which the statistical distributions suitable for describing the overall state of the system depend only on time. They are the set of functions 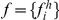, where each element 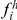 = 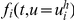 has been called a ‘discrete generalized distribution function’ corresponding to the 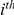 population and the 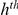 activity 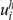. Especially, 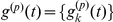 is a set of proliferating/destructive stochastic perturbation actions applied into an open bio-system from outsides, which labeled with the subscripts *k* = 1, . . . , n.

**Figure 1:**
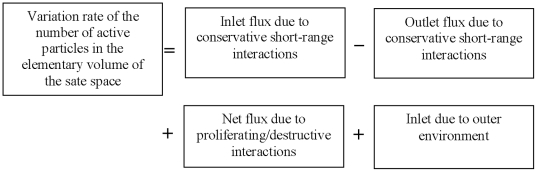
Mass balance in the state space in the case of the short-range interactions in an open bio-system.

The introduction of expression taking into account the external interactions is necessary for derivation of a general mathematical framework of an open bio-system, and the mathematical framework proposed is suitable for modeling open bio-systems of active particles including the ability to generate new particles in a population [27] [28]. The suitable balance equation can be obtained by the rate of variation of the distribution function in the elementary volume of the state space to the inlet and outlet flux due to microscopic interactions. The scheme for short-range interaction is represented in Figure 1, which is revised from [28]. The first box refers to the free transport, while the others correspond to the net fluxes in the elementary volume of the state space due to conservative and proliferating/destructive interactions, and to the inlet from the outer environment [27] [28]. Therefore, we can yield the balance equations for the discrete model. The evolution equations take the form as follows:
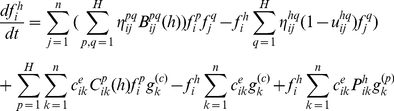
(1)where the following terms, which refer to microscopic interactions and specific actions with conservative and non-conservative external actions, have been introduced:

- The encounter rate: 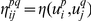, which denotes the number of encounters per unit of time between two interacting particles of the 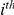 population with state 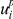 and the 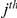 population with state 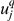.
- The discrete transition density: 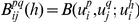, which is the probability density for a particle of the 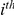 population with state 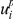 falling into the state 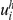 after an interaction with a field particle of the 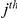 population with state 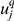.
- The source/sink rate 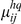, which denotes, for each encounter between a particle of the 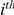 population with state 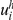 and a particle of the 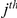 population with state 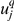, the number of particles generated or destroyed with state 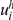 in the same population. This term is negative in the case of destructive interactions and positive for proliferating ones. Note that proliferation and/or destruction occurs with the above defined encounter rate.

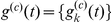
, which is a set of conservative stochastic actions labeled with the subscripts *k* = 1, . . . , n.

They are known functions of time and act over the candidate active particle of the 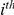 population with state 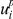. Conservative actions 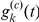 produce a transition of the particle of the 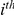 population from the state 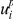 into the state 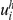.

- The external and conservative transition density function, denoted as 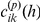, which is the probability that a particle of the 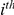 population from the state 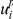 falls into the state 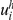 after an interaction with the 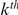 conservative external action 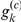.

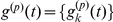
, which is a set of proliferating/destructive stochastic actions labeled with the subscripts *k* = 1, . . . , n. They are known functions of time and act over the test active particle of the 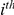 population with state 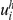. Proliferating/destructive actions 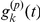 cause a proliferation or destruction of the test particle with state 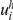.
- The external interaction rate: 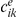, denotes external interaction rate of the candidate or test particle of the 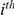 population with the 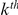 external conservative or proliferating/destructive action.
- The external and proliferating/destructive transition density function, denoted as 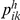, which is the proliferating or destructive rate relating to the test particle of the 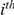 population with state 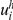, with a birth or death process in its state due to the interaction with the external non-conservative action 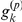.

### Model description for CRS

DNA damage, generally, can trigger two repair mechanisms in eukaryotic cells: Homologous Recombination (HR) and Non-Homologous End Joining (NHEJ) [3], [4]. Moreover, the process of DSBs repair includes a fast and a slow kinetics. The fast kinetics refers to the repair of simple DSBs, and the slow one refers to the repair of complex DSBs [5]. More specifically DSB repair is a first-order process if the break ends associated with the same DSB are rejoined, and a second-order process if the break ends associated with two different DSBs are rejoined. Although the NHEJ and HR pathways predominate in the different stages of the mammalian cell cycle, both of which may use the independent repair enzymes, HR is primarily responsible for the first-order repair process, and the NHEJ is responsible for the second-order process [4], [5].

**Figure 2:**
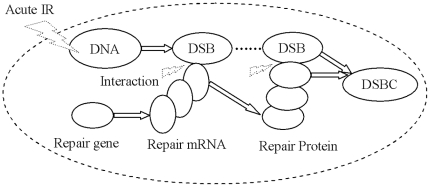
The model profile of CRS under acute IR. It is composed of two sub-populations, each of which includes some active particles with different discrete states. As acute IR is applied into a cell, the resulting DSB occur stochastically, after repair gene interacting with DSB, repair mRNA is generated, subsequently, and repair protein is generated after repair mRNA interacting with DSB. As long as RP is available around damage sites, DSBC will be synthesized after DSB combing with RP.

By the approach of KTAP, our model focuses on trying to explain the cellular self-repair mechanism under acute perturbations from outer environment. Figure 2 is the profile of CRS under acute IR. It is composed of two sub-populations, DNA damage and repair enzyme, each of which is composed of active particles with different microscopic active states. Moreover, acute IR from outer environment and molecular interactions may either modify the state of each molecular or the number of molecular in each population by proliferation/destruction phenomena.

As acute IR is applied, DNA is broken down stochastically and the resulting DSB occur into a cell [2] [3]. As a result of the interactions between repair gene and DSB, the process of repair mRNA generation is prompted in cellular response to genome stress. Meanwhile, the process of RP generation is accelerated due to the interactions between repair mRNA and DSB. Suppose RP is available around damage sites, a normal cell can start its self-repair mechanism, and then DNA damage can be synthesized into DSBC after DSB combining with RP. As a result, most of the resulting DSBs can be correctly fixed, and the correct repair part of DSBCs (rDSBCs) can transfer the damage signal into downstream gene and their regulation pathways further. Whereas, a little part of DSB can not be repaired correctly, both the disrepair part of DSBCs (mDSBCs) and the intact DSBs will be remained as toxins within a cell, which can seriously weaken the cellular viability and self-defense capability, even lead to abnormal and cancerous finally [6], [7].

### Modeling Implementation

To represent the CRS based on KTAP framework, we denote two sub-populations, including both DNA damage and repair enzyme, by two sets, 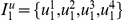 and 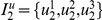, in which the elements in set 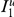 denote the active particles of DNA, DSB, rDSBC, and mDSBC, respectively, and the elements in set 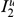 denote the active particles of repair gene, repair mRNA, and RP, respectively. Furthermore, to simplify the model, we made some following assumptions:

1. In CRS, the active particles of two sub-populations are homogeneously distributed in space undergoing localized binary interactions involving test particles and field particles, in which the test particle enters into the action domain of the field particle [28].
2. During cellular self-repair process, a fast kinetics and a slow one are involved into CRS, each of which includes a first order and a second order kinetics. More specifically, 70% of the initial DSBs is fixed by the fast repair kinetics, and the ratio of the fast rate over the slow one is chosen to be ≈10 [22]–[26].
3. Considering that Rad50/NBS1 etc nuclease complexes, a kind of repair enzyme, play an important role in both the NHEJ and the HR repair processes, therefore, we deal that the same repair enzymes are used in both repair processes [22].
4. RP can be translated from the relevant repair mRNA with basal induction rate during the DNA damage repair process, especially, the process of RP generation can be further prompted by active interactions between repair mRNA and resulting DSB [5], [22].

Based on the mathematical framework of KTAP mentioned above, we divide the CRS into several parts and implement respectively as the followings.

#### (1) DSBs generation under acute IR

**Figure 3:**
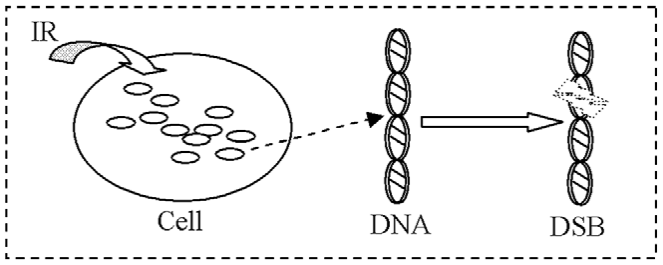
The profile of DSBs generation derived from DNA under continuous IR.

As the first part of an open CRS, the continuous function of acute IR, denoted as 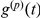, is dealt with an external action applied into a single cell from outer environment. The profile of DSBs generation induced by under continuous IR is shown in Figure 3. As external action function of acute IR perturbation, 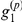, is applied into a certain cell which type is identified by 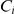, DNA is stochastically broken into two pieces of DSBs, each of which is dealt as a new DNA. Therefore, the kinetics of DSB generation and DNA increase without cellular repair mechanisms can be represent by the formulations as the followings:
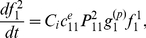
(2)and,
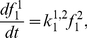
(3)where 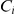 denotes the cellular type, which indicates the special destruction rate of DNA within a certain cell under external IR perturbation, 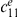 is the rate of DNA interacting with the external IR perturbation action 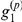, 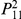 is the external and proliferating transition density function due to the interaction with the external non-conservative IR perturbation action 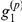, 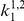 is the rate of DSB conversion into a new DNA. 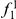, and 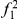 are the distribution functions of DNA and resulting DSB within a cell, respectively.

According to the fact that different number of DSBs generation in different IR dose domains, the stochastic number of resulting DSBs induced by per IR dose within each time scale obeys the principle of a Poisson random distribution, whose average is proportional to the radiation dose [2], [3], [22]–[26], we consider that, under external action function 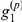, the external and proliferating transition density function 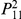 are denoted as follows:
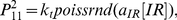
(4)Thereafter, the kinetics of DSB generation process under acute IR is rewritten by the following equation:
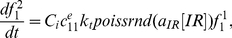
(5)where [IR] is the strength of IR dose, *k_t_* is the parameter to set the number of DSBs generation within each time scale, and *a_IR_* is the parameter to set the number of DSBs induced by per IR dose.

#### (2) RP generation process

**Figure 4:**
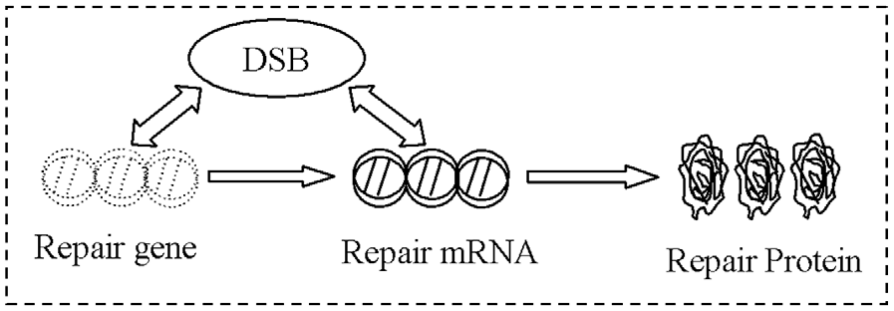
The profile of RP generation process under DSB interactions with repair gene and repair mRNA, respectively.

In our model, we deal both RP and DSB as dynamic variables, which mean that there are limited repair proteins available around increasing damage sites. More specifically, the number of RP generation depends on the quantities of initial repair gene and resulting DSBs, as well as the capability of cellular self-repair mechanisms. As shown in Figure 4, the dynamic processes of repair mRNA transcription, as well as RP generation are prompted by DSB interacting with repair gene, and repair mRNA, respectively. That is to say, the process of repair mRNA transcription is accelerated by particles interactions between DSB with state 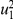 and repair gene with state 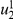, and the process of RP generation is prompted by particles interactions between DSB with state 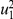 and repair mRNA with state 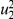. The RP generation process can be written by the following equations:
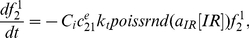
(6)

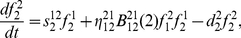
(7)

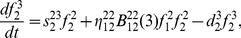
(8)where 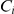 denotes the cellular type which indicate different destruction rate of repair gene within a special cell under external IR perturbation circumstances, 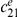 is the rate of repair gene interacting with the external IR perturbation action 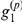, 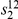 is the basal transcription rate of repair mRNA from repair gene, 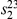 is the basal induction rate of repair protein from repair mRNA, 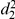 and 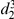 is the self-degradation rates of repair mRNA and repair protein respectively. 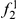, 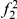 and 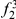 are the distribution functions of repair gene, repair mRNA and repair protein, respectively.

The second term in equation (7), 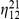, is the encounter rate between DSB in active state 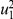 and repair gene with active state 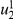, and 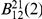 is the probability density for test particle, repair gene with state 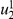, falling into the repair protein with state 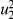 after an interaction with a field particle, DSB in state 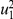. 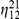 and 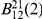 can be roughly denoted by the following equations:
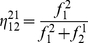
(9)

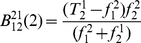
(10)where 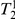 is the quantity threshold of repair mRNA generation, which indicate that the rate of repair mRNA transcription begin to decrease as the number of resulting DSB overpass 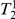.

Similarly, the second term in equation (8), 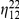, is the encounter rate between DSB with active state 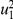 and repair mRNA with active state 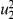, and 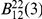 is the probability density for test active particle, repair mRNA with state 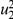, falling into the repair protein in state 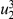 after an interaction with a field particle, DSB in state 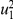. Consider the fact that RP generation is effected by the number of initial repair gene and resulting DSBs, as well as the capability of cellular damage repair mechanisms, 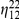 and 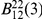 can be represent by the equations as the followings:
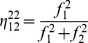
(11)

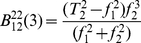
(12)where 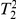 is the quantity threshold of repair protein generation, which denote that the rate of repair protein translation begin to decrease as the quantity of the resulting DSB overpass the capability threshold of cellular self-repair mechanism.

As mentioned above, the detailed framework of repair mRNA and RP generation kinetics can be rewritten as the followings:
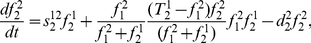
(13)

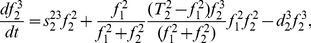
(14)


#### (3) DSBCs synthesis kinetics

**Figure 5:**
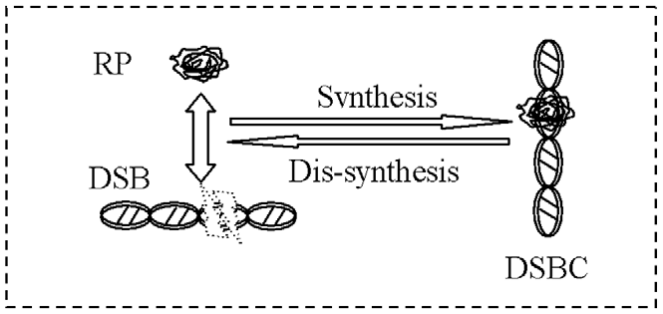
The profile of DSBC syntheses and dis-syntheses kinetics.

As mentioned above, we consider that a fast kinetics and a slow one are involved in DSBC syntheses process, each of which contains a first-order process and a second-order one. The profile of DSBC syntheses process is shown in Figure 5, in which DSB trigger the binding of RP into the nascent DNA ends, and then DSB is synthesized into DSBC. Whereas, some part of DSBC being unstable state might be reversibly broken into DSB and RP again [22]–[26]. Due to the reason that toxins including both mDSBC and intact DSB, can seriously weaken the cellular viability and self-defense capability, our model obviously distinguish mDSBC from rDSBC during DSBC synthesis processes.

Moreover, the kinetics of DSBC syntheses is determined by not only the quantity of resulting DSB, but also the number of RP available around damage sites. Especially the number of rDSBC synthesis is dealt as an indictor for reflecting the capability of a cell transferring damage signal to downstream genes and their regulation pathways, and the quantities of both mDSBC synthesis and intact DSB are dealt as an indictor for predicting genome stability in response to external perturbations [6], [7].

Similar to DSB generation process, we consider DSBC synthesis as a result of particles interactions between DSBs with active state 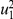, and RP with active state 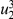. The kinetics of rDSBC synthesis can be denoted by the formulation as the followings:
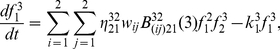
(15)and the kinetics of mDSBC synthesis is written by the following equation:
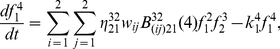
(16)where 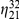 is the encounter rate between RP with active state 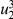 and DSB with active state 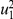. 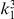, and 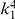 are the dis-synthesis rates from rDSBCs and mDSBCs into DSB and RP again, respectively. In different DSB repair pathways, 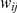 indicate the probability density for DSBC synthesis, and 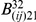 is the rate of DSBC transfer from DSB after interaction with RP, in which the subscript *i* refers to the fast and slow repair kinetics, and *j* refers to the first-order and second-order process, respectively. Thereafter, we can derive the following equations:
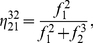
(17)

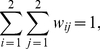
(18)

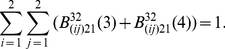
(19)


During the cellular self-repair process, the quantities of resulting DSB, RP, DSBCs and toxins within a cell are dynamically fluctuated in accordance with the strength of external IR perturbations, and different cell types, as well as the capabilities threshold of cellular self-repair mechanisms etc. Thereafter, we can derive the formulations to represent the dynamic evolution of CRS in response to acute IR perturbation circumstances. First of all, the kinetics of DSB remaining is denoted by the following equation:
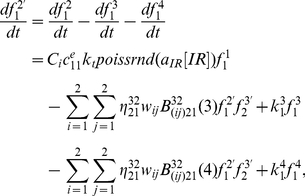
(20)and the quantities of RP available is represent by
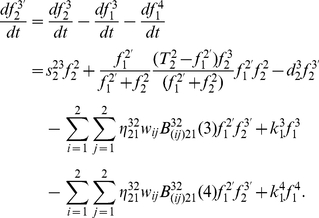
(21)


Moreover, in order to easily analyze the cellular self-repair mechanisms, we deal the quantity of RP available around damage sites as an indictor to directly predict the capability of cellular self-repair mechanisms, and the quantity of rDSBC synthesis as an indictor to predict the cellular activity of transferring genome stress into downstream gene and their regulation pathways, as well as the number of toxins accumulation within a cell as another indictor to indirectly predict cellular activity and genome stability in response to external IR perturbation circumstances. Thereafter, the kinetics of toxins accumulating and cellular activity can be denoted by the following equations:
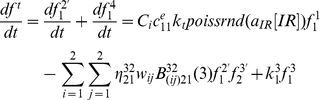
(22)

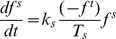
(23)where 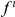 is the distribution function of toxins accumulation within a cell. 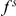 is the strength of genome stability, in which 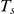 is the quantity threshold of toxins that a cell can burden maximally, and 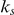 denote the rate of toxins affection on genome stability.

## Results

Based on existing studies on investigating the mechanisms of cellular response DNA damage, as well as some related parameters used in [21]–[26], we implement the kinetics of CRS on MATLAB7 platform. In the following, we simulate the dynamic kinetics of DNA damage generation, repair mRNA transcription, RP translation, DSBC synthesis and toxin accumulation. Especially, we roughly analyze the capability of cellular self-repair mechanism, and cellular activity of transferring DNA damage, as well as the of capability threshold of cellular self-repair mechanism, especially, the time thresholds of IR perturbation that a certain cell can tolerate maximally, are analyzed under different IR perturbation circumstances. The main parameters used in our simulations can be found in Table 1 of Appendix S1.

### Kinetics of DNA damage generation

In DNA damage generation process, we focus on illustrating the dynamic interactions between DNA and acute IR perturbation from outer environment, the kinetics of DSB generation, DNA damage accumulation and DNA increasing without cellular self-repair mechanisms, as well as repair gene decreasing against continuous IR perturbation. In accordance with experimental results that measured 30–40 DSBs per Gy occur as external acute IR is applied into a single cell [3], [4], [5], we consider that the initial number of resulting DSBs per time scale is proportion to the number generated by Poisson random function with a mean of 35*×*, in which *x* is strengths of IR dose [22]–[26]. Meanwhile, we deal repair gene as a part of DNA within a special cell, which means that repair gene also can be damaged by external IR. Especially, the damaged part of repair gene is dealt as DNA damage, which can not be used to generate RP again.

**Figure 6:**
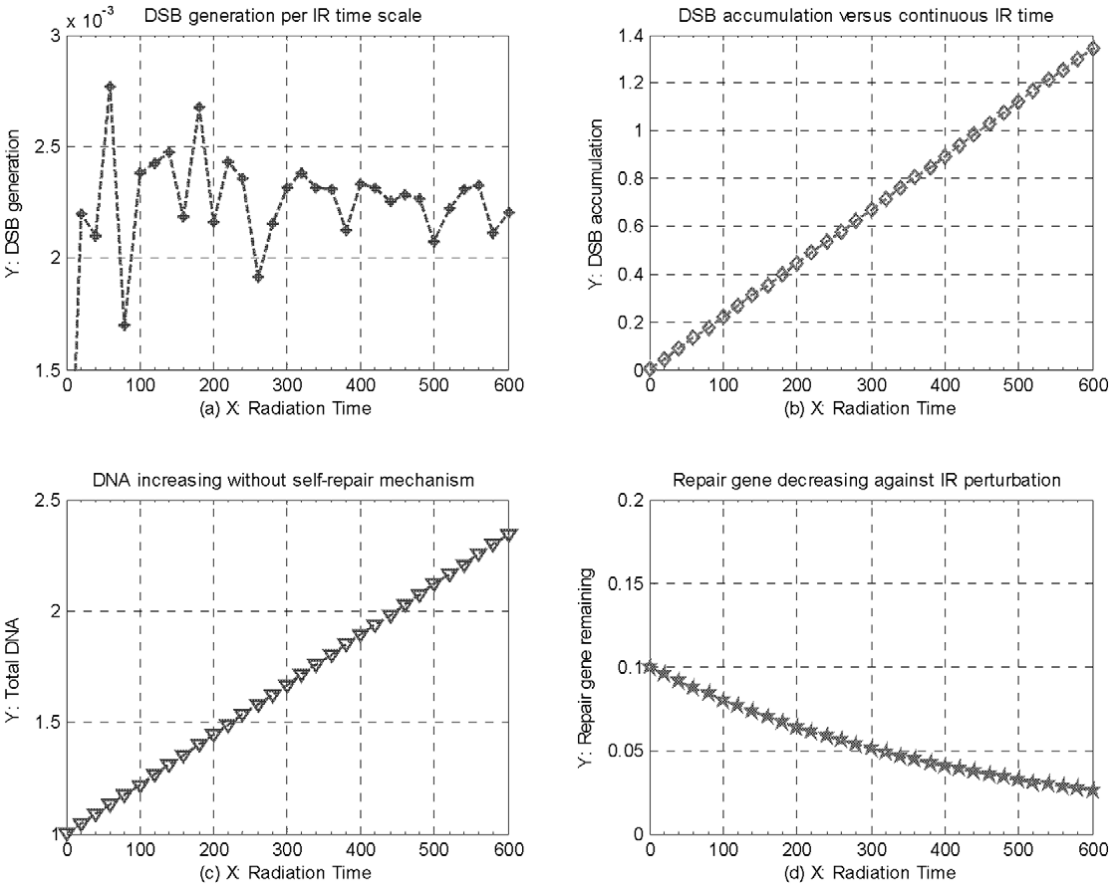
The kinetics of DNA damage generation process under constant 8Gy IR. (a) the stochastic track of DSBs generation induced by continuous 8Gy IR perturbation; (b) without cellular self-repair mechanism, the kinetics of DSBs accumulation, and (c) the kinetics of DNA increasing versus continuous IR time; (d) the kinetics of repair gene decreasing against continuous IR perturbation.

In our simulations, firstly, constant 8Gy IR is applied into a special cell, and the initial value of cell type denoted by 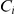 equals to 0.8, by which specify the different sensitivity and destruction rate of a certain cell in response to external IR perturbation circumstances. After interacting with external acute IR, DNA is broken down, and the resulting DSBs occur within a cell. Shown in Figure 6 (a) is the stochastic track of DSB generation per IR time scale under constant 8Gy IR. Suppose cellular self-repair mechanism is not enough or absent in a cell, as shown in Figure 6 (b), DSBs would accumulate and increase dramatically versus IR time. Meanwhile, we can see from Figure 6 (c), the number of DNA increase dramatically due to the reason that a resulting DSB is dealt as a new DNA. Moreover, as a part of total DNA within a certain cell, repair gene, plotted in figure 6 (d), begins to decrease from initial value due to the damaged repair genes increasing during interacting with external IR.

From these simulations above, we can see that the stochastic track of resulting DSB generation roughly obeys Poisson random distribution corresponding to the strength of IR dose, and is proportional with the number generated by Poisson random function with a mean of 35*×*. Especially, the kinetics of DNA damage generation above is basically in accordance with existing experimental results in [3], and previous studies in [22]–[26]. All of the simulations above suggest that, without cellular self-repair mechanisms, the genome stability within a certain cell would be broken down, and a normal cell would be destroyed by increasing DNA damage induced by continuous IR perturbation from outer environment, and lead to lethal cell death eventually.

### Kinetics of DNA damage repair

Fortunately, a normal cell can trigger its self-repair mechanism to fix up DNA damage induced by acute IR perturbation. As one of the most important steps, the kinetics of repair mRNA transcription and RP translation can provide repair enzymes to repair DSBs, and further affect the capability of cellular self-repair mechanism and genome stability of a normal cell [2]. With dynamic interactions between molecular particles, repair mRNA can be prompted to transcript from repair gene, and then RP is prompted to translate from repair mRNA. As RP is available around damage sites, DSBC can be synthesized after resulting DSB combining with RP. Therefore, the quantity of RP generation mainly affects the kinetics of DNA damage repair process.

**Figure 7:**
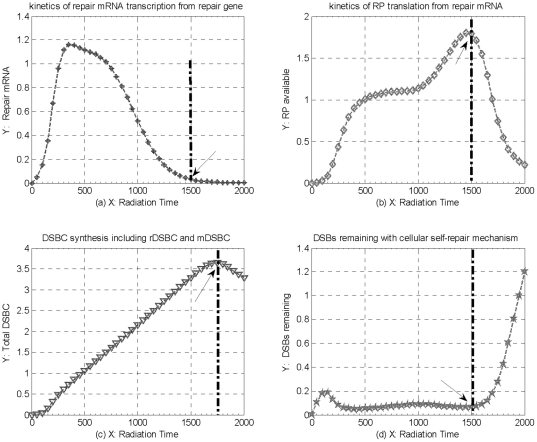
The kinetics of DNA damage repair process under constant 8Gy IR perturbation. (a) the kinetics of repair mRNA transcription from repair gene, (b) the kinetics of RP translation from repair mRNA, (c) the kinetics of DSBCs synthesis after RP combining with resulting DSBs, and (d) the kinetics of intact DSBs remaining against IR time with cellular self-repair mechanism.

Under constant 8Gy IR perturbation circumstances, the kinetics of DNA damage repair process is plotted in Figure 7. In our simulations, repair mRNA transcription is dealt as a result of particles interacting between repair gene and resulting DSB. As shown in Figure 7 (a), the number of repair mRNA transcription increases dramatically from initial value, and then begins to decrease after reaching a first climax, and finally tends to Zero after about 1500 time-scales of 8Gy IR perturbation. Meanwhile, RP is translated from repair mRNA, which is prompted by particles interacting between repair mRNA and resulting DSB. From the dynamic track of RP translation plotted in Figure 7 (b), we can see that the number of RP keep increasing with some oscillations, and then decrease sharply from a first climax after about 1500 time-scales of 8Gy IR. These simulations above indicate that, although repair mRNA and RP are dynamically consumed by the kinetics of RP generation and DSBs repair process, respectively, the quantities of repair mRNA and RP available keep increasing firstly with a little oscillations, and then reversely decrease dramatically against IR time, because of the decreasing repair gene, as well as the increasing DSBs induced by acute IR perturbation from outside.

Moreover, as a vital part of DNA damage repair process, DSBC synthesis is dealt as a result of particles interaction between resulting DSB and RP. The quantity of DSBC synthesis is affected by not only the number of resulting DSBs, but also the number of repair mRNA and RP available around damage sites. Moreover, the total number of DSBCs is split into rDSBCs and mDSBCs, the part of rDSBCs is considered as a main signal source to trigger downstream genes and their regulation pathways, and then further stimulate much more complicated cellular self-defense mechanisms in fighting against genome stresses [22]–[26]. Especially, the part of mDSBCs, apart from intact DSB, is considered as another cellular toxin, which is an unavoidable byproduct remaining within a cell during DNA damage repair process. In accordance with the track of RP available plotted in Figure 7 (b), the track of DSBC synthesis including rDSBC and mDSBC, shown in Figure 7 (c), keeps increasing straightly, and then begins to decrease after reaching a first climax due to the limited RP available around increasing DNA damage sites.

In addition, most of resulting DSBs can be repaired by cellular self-repair mechanism, whereas, a little part of DSBs still remains intact and un-repaired state. The intact DSBs are considered as a part of cellular toxins during and after the acute perturbations applied into a normal cell, which would induce serious effect on cellular activity and genome stability if this part of toxins can not be eliminated in time. As shown in Figure 7 (d), the number of intact DSBs begins to decrease after a first climax, and then keep dynamic equilibrium before 1500 time scales of 8Gy IR perturbation. Comparing simulation results plotted in Figure 6 (b) and Figure 7 (d), we can see that most of DNA damage can be repaired by cellular self-repair mechanisms as RP is available around damage sites. Unfortunately, the track of intact DSBs plotted in Figure 7 (d) begins to increase dramatically after 1500 time-scales of continuous 8Gy IR. These simulations above indicate that the quantity of RP available is indeed important to cellular self-repair mechanisms, which not only mainly reflect the capability of DNA damage repair process, but also indirectly decide cellular activity of transferring DNA damage, and genome stability as well. Especially, these simulations above suggest that a normal cell can effectively defense genome stress within a certain strength and time threshold of IR perturbation, whereas, some unavoidable byproduct, such as intact DSBs and mDSBC, will accumulate little by little, and increase dramatically after IR perturbation overpass a certain time threshold, and eventually induce much more serious consequence, such as cell abnormal or lethal cell death, if the complicated cellular degradation mechanism can not be triggered further.

### Analysis for the cellular activity, repair capability, and genome stability

**Figure 8:**
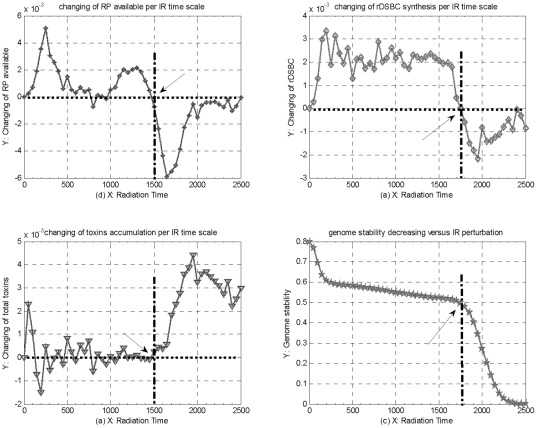
Analysis for capability of cellular self-repair mechanism, cellular activity and genome stability under constant 8Gy IR perturbation. (a) the track of RP available changing, (b) the track of rDSBC synthesis changing, and (c) the track of toxins accumulation changing per IR time-scale of continuous 8Gy IR perturbation, as well as (d) the track of genome stability accordingly decreasing versus IR perturbation time.

As mentioned above, RP generation is one of the most vital steps in DNA damage repair process, and the kinetics of DNA damage repair process mainly depends upon the quantities of mRNA generation and RP available. Therefore, we deal the kinetics of RP available as an indicator to predict the capability of cellular self-repair mechanism. As shown in Figure 8 (a), under constant 8Gy IR perturbation, the track of RP available is dynamically changed within each time-scale, from which we can see that RP available positively increase before 1500 time- scales of 8Gy IR, and then begin to negatively decrease sharply after IR time overpass 1500 time-scales, and finally tend to dynamic equilibrium versus continuous IR time. This simulations indicate that, in accordance with the track of RP available changing, the capability of cellular self-repair mechanism begins to decrease sharply and then tends to a certain minimal value after IR perturbation overtakes 1500 time-scales of constant 8Gy IR.

Also, rDSBC synthesis is a key product in both fast and slow DNA damage repair processes, and the quantity of rDSBC synthesis can be dealt as a main signal source to stimulate downstream genes and their regulation pathways, and then trigger much more complicated cellular degradation mechanism which can effectively eliminate toxins within a cell further [21]. Therefore, we deal the kinetics of rDSBC synthesis as an indicator to analyze the cellular activity of transferring DNA damage signal to trigger much more complicated cellular defending mechanisms in fighting against genome stress. As shown in Figure 8 (b), the track of rDSBC synthesis changing per time-scale positively increases before about 1750 time-scales of constant 8Gy IR perturbation, and then negatively decreases and eventually tends to a dynamic equilibrium versus continuous IR time. This simulation result above suggest that cellular activity of transferring DNA damage begins to decrease as IR perturbation overpass a certain IR time threshold, which is similar with the kinetics of RP changing plotted in Figure 8 (a), apart from with about 150 time-scales delay in the latter.

Meanwhile, suppose cellular degradation mechanism is not enough or absent, cellular toxins including intact DSBs and mDSBC, would remain and accumulate little by little within a cell, which can weaken cellular activity and genome stability, and further lead into cell abnormal, even lethal cell death eventually [6], [7]. Therefore, we deal both mDSBCs and intact DSBs as an indictor to roughly analyze genome stability in response to acute IR perturbation. As shown in Figure 8 (c), the track of cellular toxins changing per time-scale tends to dynamic equilibrium with decreasing oscillations before 1500 time-scales of 8Gy IR perturbation, and then begins to positively increase dramatically against continuous IR time. As a result, the track of genome stability, shown in Figure 8 (d), begins to sharply decrease from initial value, and then keep decreasing slowly after about 200 time-scales of 8Gy IR, finally, the track begins to quickly decrease after 1750 time-scales and tends to Zero against continuous IR perturbation.

These simulations indicate that without cellular degradation mechanism, genome stability would keep decreasing, especially, the genome stability would be broken down, and then a normal cell would be damaged further. Especially, suppose IR perturbation overpasses a certain time threshold of IR perturbation domain that a normal cell can tolerate maximally, cellular self-repair mechanism would be destroyed, and then genome stability would be broken down, a lethal cell death would occur eventually.

### Cellular Self-Repair Mechanisms under Different IR Perturbation Circumstances

In order to investigate different kinetics of cellular self-repair mechanisms in response to different IR perturbation circumstances, we illustrate the different kinetics of RP translation, rDSBC synthesis, and further analyze the different capabilities of cellular self-repair mechanisms, cellular activities of transferring DNA damage, and genome stabilities in response to different toxins accumulation, respectively. Especially, the time thresholds of different IR perturbations which a certain cell can tolerate maximally are analyzed by comparing different simulation results.

**Figure 9:**
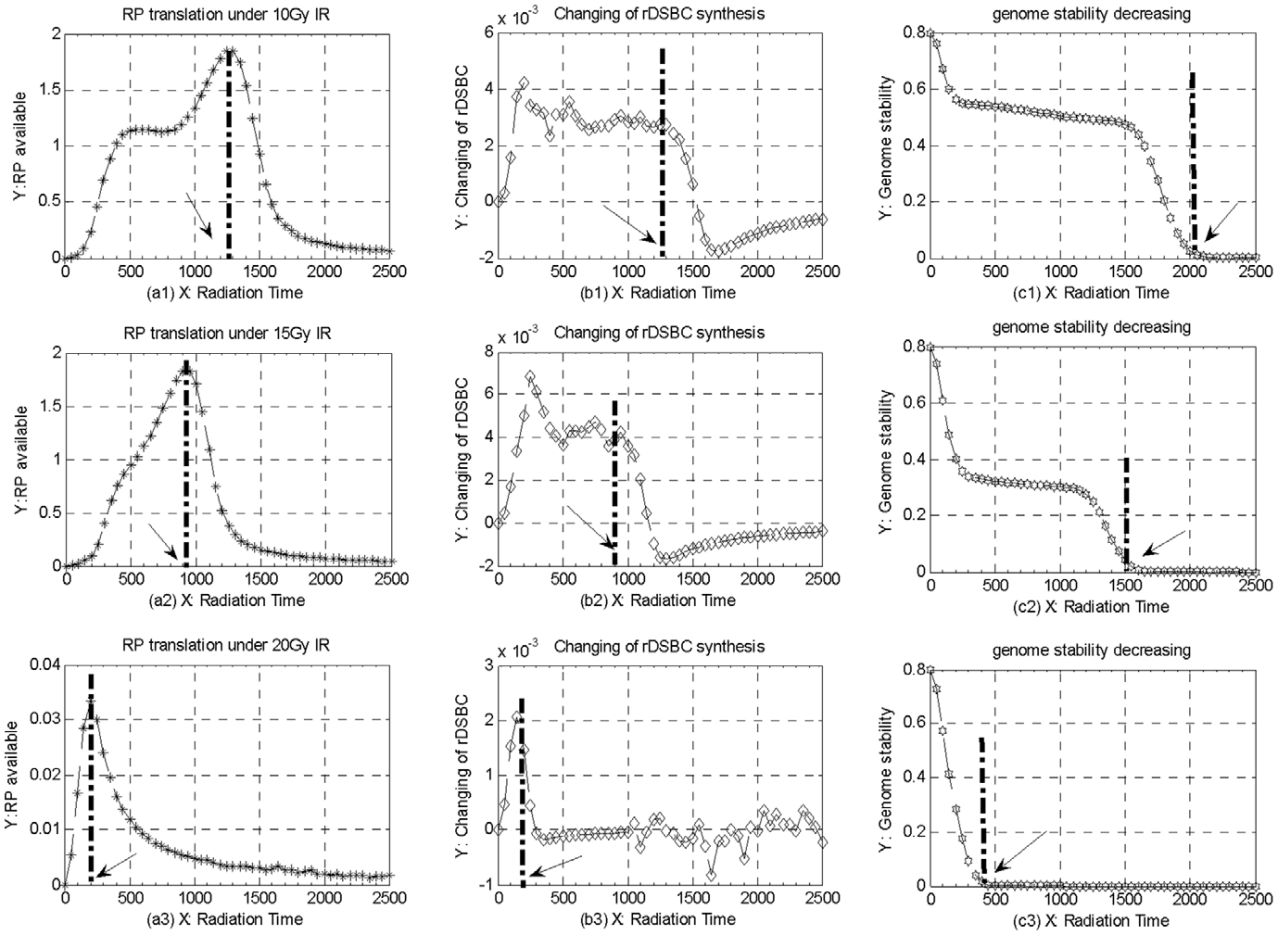
The different kinetics of cellular self-repair mechanisms under different 10, 15, and 20Gy IR, respectively. (a1),(a2),(a3) the different tracks of RP translation processes from repair mRNA; (b1),(b2),(b3) the different tracks of rDSBC synthesis changing per time-scale according to different tracks of RP available; (c1),(c2),(c3) the different tracks of genome stability decreasing in accordance with different kinetics of toxins accumulation under different IR perturbations.

As mentioned above, RP available is vital for cellular self-repair mechanism within a certain cell, which directly decides dynamic kinetics of DSBC synthesis and toxins accumulation, as well as the capability of DNA damage repair process especially. Suppose a normal cell is exposed into different 10, 15, and 20Gy, respectively, as shown in Figure 9 (a1),(a2),(a3), the quantities of RP available begin to decrease dramatically from different concentration climaxes, and then tend to dynamic equilibriums versus different IR perturbations. These simulations above suggest that the capability of cellular self-repair mechanism begin to decrease after reaching a certain time threshold of IR perturbation due to much less RP available around increasing DNA damage sites, especially, this capability would become much weaker and keep much shorter time as much stronger IR perturbation is applied into a cell.

Meanwhile, we deal that the quantity of rDSBCs synthesis indirectly denotes the cellular activity of transferring DNA damage in response to genome stress. As shown in Figure 9 (b1),(b2),(b3), under different 10, 15, and 20Gy IR, the dynamic tracks of rDSBCs changing per time-scale indicate that much less rDSBCs can be synthesized, and much less time is need to reach the dynamic equilibriums due to much less RP available around increasing damage sites. Theses simulations above suggest that, the cellular activity and capability of transferring DNA damage keep deceasing and tend to Zero as IR perturbation overtakes a certain time threshold that a normal cell can endure maximally, especially, the cellular activity become much weaker as much stronger IR and much longer perturbation time is applied into a normal cell. Moreover, cellular toxins including both intact DSBs and mDSBCs can seriously weaken the genome stability little by little without cellular degradation mechanism. As shown in Figure 9 (c1),(c2),(c3), in response to 10, 15, and 20Gy IR perturbation circumstances, the different kinetics of genome stabilities indicate that genome stability would keep decreasing and tend to Zero after IR perturbations overtake their respective time thresholds, which means that a normal cell would tend to fall into irreversible cell death if genome stability is broken down after IR perturbation exceed a certain time threshold.

All of these simulation results above suggest that, within special time threshold of a certain strength IR perturbation, a normal cell can start its self-repair mechanisms, and fix up the resulting DNA damage. Furthermore, a cell can relay genome stress signal to downstream genes and their regulation pathways, and then trigger much more complicated self-defense mechanisms, such as toxins degradation kinetics, and try to survive itself from genome stress induced by IR perturbation environment. Unfortunately, suppose IR perturbation overtakes a certain time threshold that a cell can burden maximally, RP available would sharply decrease, and then cellular toxins accumulate and increase dramatically within a cell. Consequently, cellular self-repair capability, cellular activity and capability of transferring DNA damage signal, as well as genome stability would decrease in accordance with the perturbation strength and perturbation time of IR dose domains from outsides. Especially, much stronger IR is applied, much shorter time is spent to make a normal cell break down, and more seriously, even lead to lethal cell death eventually.

## Discussion

Under acute IR perturbation from outer environment, a feasible model is proposed to investigate cellular self-repair mechanism at single cell level. Based on mathematical framework of KTAP, our model illustrates the dynamic kinetics of DSB generation, and repair mRNA transcription and RP translation, as well as DSBC synthesis by using particle interactions between a pair of molecules within different populations. The process of DSB generation, the first part of the model, denotes a stochastic kinetics of DNA damage occurrence induced by external IR perturbation, and provides a signal source to stimulate cellular self-repair mechanism further. As a vital part of DNA damage repair process, RP translation from repair mRNA are considered as one of the most important steps in cellular self-repair mechanism, especially, the number of RP available directly affect the capability of DNA damage repair process. In the process of DSBC synthesis, rDSBCs synthesized by resulting DSBs and RP, can transfer DNA damage signal into downstream genes and their regulation pathways, and then trigger much more complicated cellular self-defense mechanisms to fight against genome stress further. Whereas, as byproducts of DNA damage repair processes, cellular toxins including intact DSBs and mDSBCs would accumulate and increase little by little if cellular eliminating functions are not enough or absent, which can further weaken cellular activity and genome stability, even lead to more serious cell death eventually. Especially, the quantity of RP are dealt as an indicator for predicting capability of cellular self-repair mechanisms, the number of rDSBCs synthesis are considered as an indicator for indicting cellular activity of transferring DNA damage signal and triggering much more complicated self-defending mechanism further, and the quantity of toxins accumulation can be dealt as an indictor for indirectly denoting genome stability under continuous IR perturbation.

In our simulations, firstly, we implement the dynamic kinetics of cellular self-repair processes including DSB generation, mRNA and RP generation, DSBC synthesis, and cellular toxins accumulation under 8Gy IR; secondly, we analyze the capability of cellular self-repair mechanism, cellular activity and capability of transferring DNA damage, as well as genome stability in response to continuous 8Gy IR; finally, we illustrate different cellular self-repair mechanisms by comparing different dynamic kinetics of RP generation, rDSBC synthesis, and genome stability under different 10, 15, and 20Gy IR perturbations, respectively. The simulation results indicate that a normal cell can trigger self-repair mechanism to fix up DNA damage within a certain time threshold of IR perturbation. Unfortunately, if IR perturbation overtakes a certain time threshold that a cell can burden maximally, cellular self-repair capability, cellular activity and genome stability would decrease quickly, and then a lethal consequence of cell abnormality and cell death occur eventually. Especially, suppose much more strength of IR perturbation is applied into a normal cell, much shorter time would be spent to reach dynamic equilibriums, and much more serious fates a cell would encounter, such as cell abnormal, even lethal cell death eventually. Our model, although simple, is flexible and suitable for investigate the simulation of DNA damage repair processes by through complicated interactions between molecular particles, and dose provide a mathematical framework to analyze the capability of cellular self-repair mechanism, cellular activity of transferring genome stress, genome stability in response to toxins accumulation, as well as time threshold that a certain cell can tolerate maximally under different continuous IR perturbations from outer environment.

## Supporting Information

Appendix S1 Table 1. The main parameters used in the kinetics of cellular self-repair mechanism.
(DOC)
