# Correction: Kinetic Theory Approach to Modeling of Cellular Repair Mechanisms under Genome Stress

**DOI:** 10.1371/annotation/7a803b23-f447-4676-a795-8b1b54f4d95d

**Published:** 2011-09-23

**Authors:** Jinpeng Qi, Yongsheng Ding, Ying Zhu, Yizhi Wu

The authors would like to replace this existing funding information with the following: "The latest information of founds is that "This work is supported in part by the National Nature Science Foundation of China (No.61104154, No.11102038 and No.60975059), Specialized Research Fund for the Doctoral Program of Higher Education from Ministry of Education of China (No. 20090075110002), Specialized Research Fund for Natural Science Foundation of Shanghai (No.10ZR1401600), Project of the Shanghai Committee of Science and Technology (No. 10JC1400200), and Shanghai Education Development Foundation Chenguang Project (No.10CG33)." 

