# Supplementary material for: Kinetic Theory Approach to Modeling of Cellular Repair Mechanisms under Genome Stress
Source: PLoS One. 2011 Aug 9;6(8):e22228. doi: 10.1371/journal.pone.0022228 (PMC3153456; doi:10.1371/journal.pone.0022228)
Supplement: Appendix S1 — Table 1. The main parameters used in the kinetics of cellular self-repair mechanism. (DOC) [file pone.0022228.s001.doc]

**Appendix**

Tab 1. The main parameters used in the kinetics of cellular self-repair mechanism

| Parameters | Description | Constant |
| --- | --- | --- |
| *C_i_* | The cell type which denote the destruction rate from 0 to 1 | 0.8 |
| $c_{11}^{e}$ | The interaction rate of DNA with external perturbation | 1 |
| $k_{1}^{1,2}$ | The rate of resulting DSB conversion into a new DNA | 1 |
| *k_t_* | The rate of DSBs generation per time scale | 0.001 |
| *a_IR_* | The number of DSBs generation per IR dose | 35 |
| $c_{21}^{e}$ | The interaction rate of repair gene with external IR | 1 |
| $s_{2}^{12}$ | The basal transcription rate of repair mRNA from repair gene | 0.01 |
| $s_{2}^{23}$ | The basal transcription rate of RP from repair mRNA | 0.01 |
| $d_{2}^{2}$ | The self-degradation rate of repair mRNA | 0.001 |
| $d_{2}^{3}$ | The self-degradation rate of RP | 0.001 |
| $T_{2}^{1}$ | The quantity threshold of repair mRNA generation decreasing | 0.6 |
| $T_{2}^{2}$ | The quantity threshold of RP generation decreasing | 0.6 |
| $k_{1}^{3}$ | The dis-syntheis rate from rDSBC into DSB and RP | 0.01 |
| $k_{1}^{4}$ | The dis-syntheis rate from mDSBC into DSB and RP | 0.005 |
| $B_{(ij)21}^{32}$ | The rate of rDSBC synthesis after DSB interacting with RP | 0.99 |
| $B_{(ij)21}^{42}$ | The rate of mDSBC synthesis after DSB interacting with RP | 0.01 |
| $T_{s}$ | The quantity threshold of toxins that a certain cell burden maximally | 0.2 |
| $k_{s}$ | The rate of toxins affection on genome stability | 0.5 |
